# Genetic Signature of *Pinctada fucata* Inferred from Population Genomics: Source Tracking of the Invasion in Mischief Reef of Nansha Islands

**DOI:** 10.3390/biology12010097

**Published:** 2023-01-09

**Authors:** Binbin Shan, Gang Yu, Liangming Wang, Yan Liu, Changping Yang, Manting Liu, Dianrong Sun

**Affiliations:** 1 Tropical Aquaculture Research and Development Center, South China Sea Fisheries Research Institute, Chinese Academy of Fishery Sciences, Sanya 572000, China; 2 Key Laboratory of Marine Ranching, Ministry of Agriculture Rural Affairs, Guangzhou 510300, China; 3 South China Sea Fisheries Research Institute, Chinese Academy of Fisheries Sciences, Guangzhou 510300, China

**Keywords:** Mischief Reef, *Pinctada fucata*, introduced population, geographic origin, population genetics

## Abstract

**Simple Summary:**

In the present study, we employed population genetics analysis to investigate possible origins of the introduced *Pinctada fucata* population in the Mischief Reef of the South China Sea, as well as diversity and structure of the *P. fucata* populations. Population genomics data clearly revealed a closer genetic relationship between the Mischief Reef introduced population and the Lingshui population, indicating that Lingshui may be the potential geographical origin. Furthermore, several selected genomic regions and genes of the introduced population were identified, some of which may play important roles in the adaptation of temperature and salinity tolerance.

**Abstract:**

Among the anthropogenic stresses that marine ecosystems face, biological invasions are one of the major threats. Recently, as a result of increasingly intense anthropogenic disturbance, numerous marine species have been introduced to their non-native ranges. However, many introduced species have uncertain original sources. This prevents the design and establishment of methods for controlling or preventing these introduced species. In the present study, genomic sequencing and population genetic analysis were performed to detect the geographic origin of the introduced *Pinctada fucata* population in the Mischief Reef of the South China Sea. The results of population genetic structure analysis showed a close relationship between the Mischief Reef introduced population and the Lingshui population, indicating that Lingshui may be the potential geographical origin. Furthermore, lower heterozygosity and nucleotide diversity were observed in the introduced population in Mischief Reef, indicating lower genetic diversity than in other native populations. We also identified some selected genomic regions and genes of the introduced population, including genes related to temperature and salinity tolerance. These genes may play important roles in the adaptation of the introduced population. Our study will improve our understanding of the invasion history of the *P*. *fucata* population. Furthermore, the results of the present study will also facilitate further control and prevention of invasion in Mischief Reef, South China Sea.

## 1. Introduction

Marine ecosystems are threatened by a number of anthropogenic stresses, including overexploitation of living resources, pollution, climate change, biological invasion and others [1]. Numerous studies have documented that, among these pressures, bioinvasion is one of the major threats that may cause significant changes and degradation of marine biodiversity [2,3]. In general, a biological invasion occurs when species are outside their native range, expanding their original ecological spaces and impacting local biodiversity and resources [4]. In recent decades, marine bioinvasions have become increasingly frequent as a result of anthropogenic disturbance, leading to species translocations through aquaculture, hull fouling, ballast water, etc. [5,6]. The introduction of invasive aquatic species into new habitats has been identified as one of the greatest threats to the world’s oceans [7]. Given the accelerating global threat to marine ecosystems, researchers and managers are increasingly focused on tracking, preventing and managing invasions.

Pearl oysters belonging to the genus *Pinctada* (Bivalvia: Pteriidae) are widely distributed between the Indo-Pacific and Western Atlantic tropical and subtropical shallow-water areas, most of which are associated with reef environments [8]. *Pinctada fucata* (Gould, 1850), also named *P. martensii*, is an economically important bivalve native to the coastal waters of the Tropic of Cancer and the Tropic of Capricorn in the Pacific Ocean [9]. It is predominantly cultured for pearl production in China, Japan and Korea [10,11,12].

**Figure 1:**
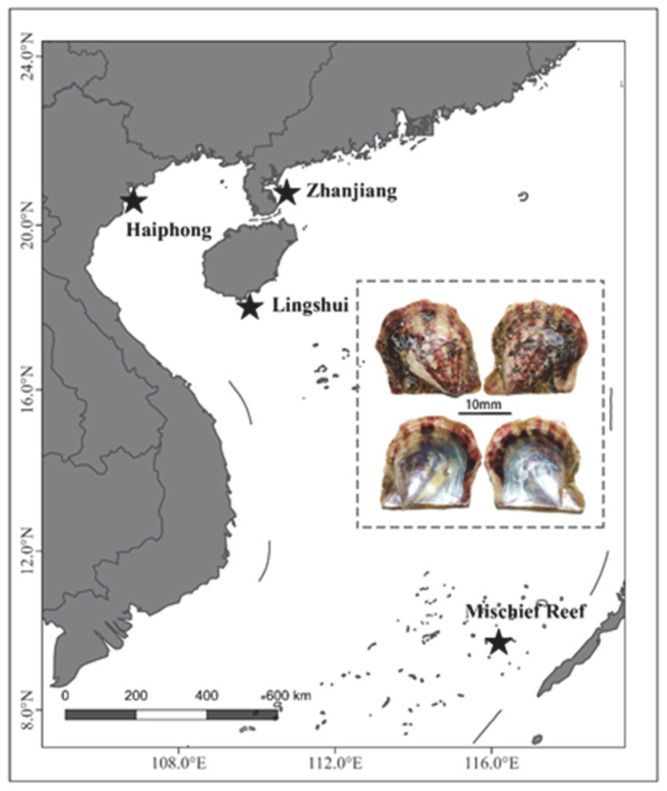
The sample sites in the present study. Stars represent the sampling sites.

Mischief Reef is located in the eastern Nansha Islands of China (Figure 1). In recent decades, with increased development in this area (reclamation, aquaculture and fishery), another species of *Pinctada* can be found in this area. Wang [13] and Chen [14] reported that *Pinctada maculata* was a unique species of the genus *Pinctada* in the Nansha Islands sea area. However, during a recent sampling at Mischief Reef, *P*. *fucata* was found in extreme abundance. According to the local fishermen, the occurrence of *P*. *fucata* in Mischief first occurred in 2016 (Figure S1). Even though Mischief Reef is within the theoretical distribution range of *P. maculata*, it is still a non native speices. Similar to many other notable invaders, *P*. *fucata* has not caused significant ecological or economic problems in its native area. However, as a potential invader, it is unknown whether the introduction and potential subsequent spread of *P*. *fucata* will damage the ecosystem of Mischief Reef or not.

In the management of marine ecosystems, reconstructing the complex history of invasions is essential and plays a crucial role in preventing subsequent spread [15,16]. A crucial piece of information in the reconstruction of the invasion history is the investigation of invaders’ geographical origin [17]. Furthermore, if the sources and pathways responsible for the introduction could be investigated, then a monitoring and control strategy could be established [3]. However, in practice, such investigation is extremely difficult via ecological surveys or other observational approaches. For bivalve species, their soft tissues and shell morphology are not suitable for identifying source populations or cryptogenic taxa [18].

The development and common application of molecular markers in evolutionary and ecological studies has facilitated the tracking of original sources of these invaders, sometimes with surprising precision [16,19]. Recent years have seen a dramatic rise in the tracing of the biogeographical history of many invasive species in marine systems [20,21,22]. For instance, combined population genetic and phylogeographic analyses provided evidence of multiple complex invasions of the European green crab *Carcinus maenas* on almost every continent with temperate shores [20]. Another notable case was the invasion of the Pacific oyster *Crassostrea gigas* in NW Europe. Molecular approaches have been effectively utilized when exploring the invasion histories of Pacific oysters [22].

In the present study, we used a population genomics approach utilizing restriction-site associated DNA (RAD) sequencing and comparative genomics to detect the geographic origin of invasive *P*. *fucata* in Mischief Reef, describing the present invasive population’s genomic diversity. Furthermore, we aimed to identify selected genomic regions and genes of the introduced population of *P*. *fucata* and identify some genes related to environmental adaptation. Our study will improve understanding of the invasion history of *P*. *fucata* in Mischief Reef.

## 2. Materials and Methods

### 2.1. Sampling and Sequencing

From 2017 to 2018, a total of 74 *P. fucata* individuals were obtained from four geographic sites, including the native range (Zhanjiang, *n* = 24; Lingshui, *n* = 14; Haiphong, *n* = 18) and non-native range (Mischief Reef, *n* = 18) (Figure 1). All specimens were frozen at −80 °C and genomic DNA was extracted from adductor muscle using a TIANGEN TIANamp Marine Animals DNA Kit. Briefly, the RAD-seq library was prepared following the protocol after DNA quality assessment [23]. Then, sequencing was performed on the BGISEQ-500RS (BGI, Shenzhen, China) using a 100-bp paired-end strategy.

### 2.2. Alignment and Variation Calling

BWA-mem (version: 0.7.17, https://github.com/lh3/bwa, accessed on 24 October 2017) was applied to align the clean reads (default parameters) to the *P. fucata* reference genome sequence [24]. Then, these reads were sorted by the SAMtools “sort” function (version: 1.9, https://github.com/samtools/samtools/releases, accessed on 19 July 2018) [25]. GATK tools version 4.1.2.0 (BaseRecalibrator, HaplotypeCaller and CombineGVCFs, https://github.com/broadinstitute/gatk/releases, accessed on 24 April 2019) was utilized to identify and call high-quality SNPs, with refinement stages similar to those of Todesco et al. [26,27]. Finally, GATK-VariantFiltration was used to filter high-quality SNPs by the following criteria: MQ < 40.0, QD < 2.0, FS > 60.0, SOR > 12.0, MQRankSum < −12.5, ReadPosRankSum < −8.0.

### 2.3. Population Genetics Analysis

For all *P. fucata* populations, *θ_w_*, *θ_π_* and Tajima’s *D* values were calculated for the assessment of genetic diversity using a sliding window approach and the step and window sizes were set as 5 kb and 10 kb, respectively [28,29]. To measure the differentiation between the four *P. fucata* populations pairwise, the fixation index (*F_ST_*) values were also calculated in the same windows [30]. We assessed the genetic relationships between the *P. fucata* population from Mischief Reef and other populations. Principal component analysis was conducted via PLINK (version 2.0, www.cog-genomics.org/plink/2.0/, accessed on 29 January 2022) and GCTA (version 1.93.0, https://yanglab.westlake.edu.cn/software/gcta/#Download, accessed on 9 December 2019) [31]. To estimate the genetic diversity of the *P. fucata* population, PLINK (version 2.0) was also used to calculate the expected heterozygosity (*H_e_*) and observed heterozygosity (*H_o_*). Furthermore, admixture analysis was performed to detect population structure by using ADMIXTURE (version: 1.3.0) [32]. The number of ancestral clusters was assumed to range from 2 to 10.

We also evaluated the genetic connectivity between the four populations by examining the gene flow between them. The analysis was performed based on the directional migration method by estimating the relative migration rates derived from *G_st_*, *N_m_* and *D* [33,34,35]. The relative migration rates and network were determined by using the function divMigrate from the R package diveRsity, with a bootstrap value of 1000 and a filter threshold of 0.2 [36].

Furthermore, we used VCF2Dis (version 1.47, https://github.com/BGI-shenzhen/VCF2Dis, accessed on 25 July 2022) to calculate the pairwise genetic distances (*p* distance). Then, PHYLIPNEW (version 3.69, http://evolution.genetics.washington.edu/phylip.html, accessed on 2 October 2019) was utilized to construct an NJ tree (Neighbour-Joining tree). The squared Pearson’s correlation coefficient (*r*^2^) was calculated to evaluate the linkage disequilibrium (LD) in four *P. fucata* populations. The analysis was performed by using PopLDdecay version 3.40 (https://github.com/BGI-shenzhen/PopLDdecay/releases, accessed on 16 January 2019) [37]. Then, we measured LD decay based on the distance of each pair of SNPs and *r*^2^ values.

### 2.4. Potential Selected Regions in the Introduced Population

To identify selected genomic regions and genes of the introduced population of *P. fucata*, we screened the SNPs called across the introduced population and its potential original population (Lingshui population). Then, we estimated pairwise differentiation between the two populations and Tajima’s *D*, *F_ST_* and ROD (1 − *θ_π-Mischief_*/*θ_π-Lingshui_*) were calculated by using a sliding window approach. The window size was 10 kb and the step size was 5 kb. In our study, an empirical threshold of 5% was set to identify outlier windows of *F_ST_* (top 5%), Tajima’s *D* (bottom 5%) and ROD (top 5%) values. The outlier windows were considered candidate regions for selection with the following criteria: the top 5% of *F_ST_* values or the top 5% of ROD values with the bottom 5% of negative Tajima’s *D* values.

## 3. Results

### 3.1. Variant Calling and Population Genetic Diversity

With the sequencing of 74 samples from four sites (Figure 1), we generated a total of 1207.53 million reads (16.32 million reads per sample) after quality filtering (Supplementary Table S1). Then, all clean reads were mapped against the *Pinctada martensii* reference genome (ftp://parrot.genomics.cn/gigadb/pub/10.5524/100001_101000/100240/, accessed on 18 October 2021). The average mapping rate was 94.89% (range from 93.63% to 95.15%). In the present study, we selected the top 30 samples with the highest sequencing coverage for calibration. SNPs with a missing rate less than 50% and MAF greater than 0.01 were utilized for the downstream analysis. Then, we obtained 3,476,259 SNPs. Of these SNPs, 254,373 SNPs (7.32%) were aligned to exonic regions, 1,017,991 SNPs (29.28%) were aligned to intronic regions and 1,956,307 SNPs (56.28%) were aligned to intergenic regions. Among the 254,373 SNPs in exonic regions, 100,402 were nonsynonymous and 147,749 were synonymous.

**Figure 2:**
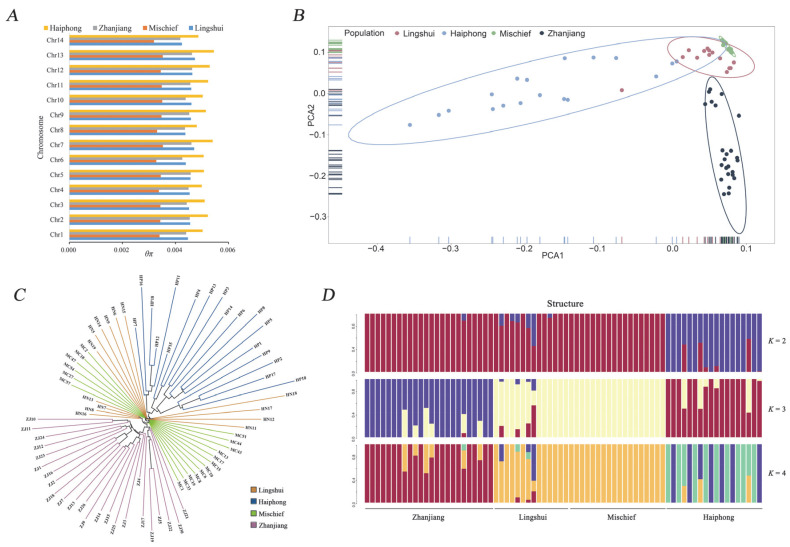
Population genetic diversity and structure of four *P. fucata* populations. (**A**). *θ_π_* values of different chromosomes of the four *P. fucata* populations. (**B**). PCA plot of the first two principal components based on all SNPs. (**C**). The neighbor-joining phylogenetic tree of all samples. (**D**). The structure analysis graph under different *K* values.

The genetic diversity of the four populations was assessed by estimating the nucleotide diversity. As shown in Table 1, the Lingshui and Haiphong populations exhibited the highest genetic diversity, both with *θ_w_* and *θ_π_* values of 0.005. Among the four populations, the *θ_w_* and *θ_π_* of the nonlocal population (Mischief Reef) were lowest, showing a lower gene diversity than in the other three populations (Figure 2A). The results of heterozygosity showed the highest genetic diversity in the Haiphong population and the lowest genetic diversity in the Mischief Reef population.

### 3.2. Population Genetic Structure

The results of PCA showed a clear genetic relationship among the four *P. fucata* populations (Figure 2B). The top three principal components (PCs) explained 4.62%, 3.19% and 2.94% of the variation, respectively. In the plot, the populations from Lingshui and Mischief Reef were clustered into a group and those from Zhanjiang and Haiphong were classified into two separate groups. In addition, an NJ phylogenetic tree was constructed (Figure 2C). The topological structure of the tree showed that the Mischief Reef population was more similar to the Lingshui population than the other two populations, indicating a close relationship between the Lingshui population and the introduced population at Mischief Reef.

To further investigate the relationships between the introduced population and other populations, we performed admixture analysis with an assumed number of ancestral clusters from two to six (Figure 2D). The results showed that the cross-validation error (CV error) reached the smallest value when the ancestral cluster number was set as two (Figure S2). However, we considered the estimated cluster number (*K* = 2) unsuitable for two reasons. First, only Haiphong clearly formed an independent group and the introduced population in Mischief Reef was undistinguishable from the Lingshui and Zhanjiang populations. Furthermore, the CV error gap between *K* = 2 and *K* = 3 was not very significant and the second-order rate of change increased after *K* = 3. Thus, the clear genetic relationship when the ancestral cluster number was three provides a more biologically suitable argument than the more conservative number. The fixation index (*F_ST_*) was then calculated to measure the genetic difference between the Mischief Reef population and other local populations (Table 2), indicating a closer genetic relationship between the two populations than others.

**Figure 3:**
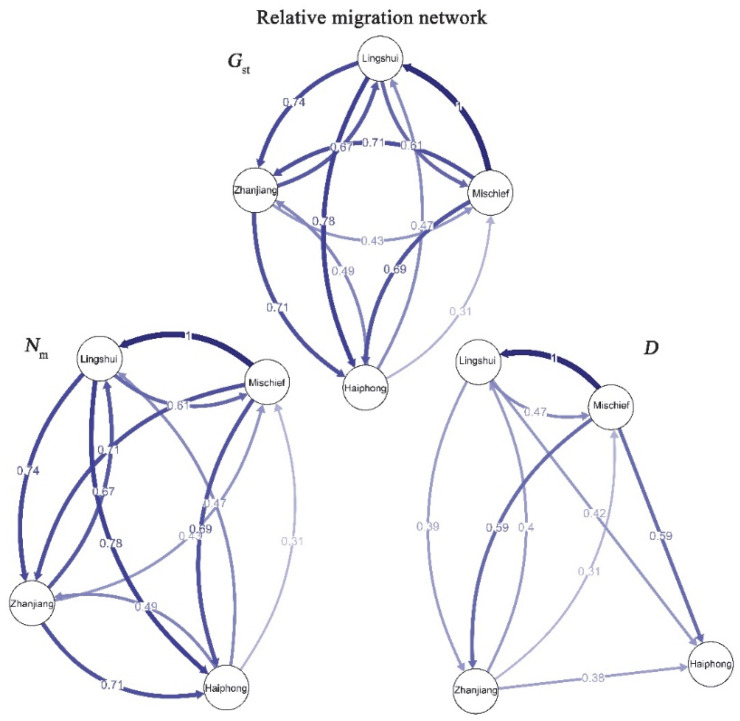
Relative migration network among the four populations based on different index.

The relative migration network constructed based on divMigrate analysis indicated gene flow between the four *P. fucata* populations (Figure 3). High and moderate gene flow between the Mischief Reef, Zhanjiang and Lingshui populations were observed. Gene flow between Haiphong and other populations was weak. Among these populations, the Mischief Reef and Lingshui populations showed the strongest migration rates, indicating a closer relationship than between the other populations. However, no strong directionality could be detected.

**Figure 4:**
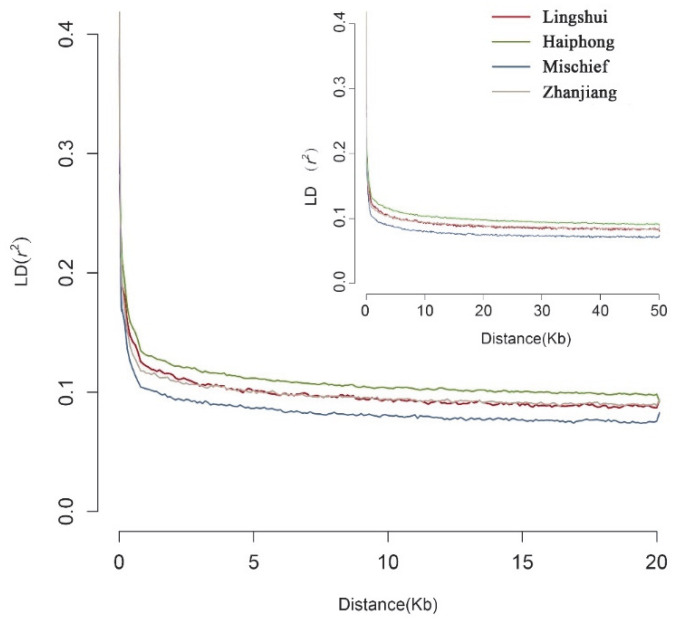
Linkage-disequilibrium decay for different distances of the four populations.

The bottleneck effect can greatly change the allele frequency of sites in the population, which is the main reason for the drastic change in Linkage disequilibrium (LD) in a short time [38]. The results of linkage disequilibrium analysis showed that, both over a longer distance (≥20 Kb) and over a short distance (≤20 Kb), the Mischief Reef population had the lowest *r*^2^ value with the fastest LD decay rate among the four populations (Figure 4). Meanwhile, the Haiphong population had the slowest decay rate, indicating potential artificial domestication.

### 3.3. Population Genetic Structure

In the present study, the values of Tajima’s *D* for populations were significantly different. Among these populations, only the Tajima’s *D* of the Zhanjiang population was close to zero. For the Lingshui population and Mischief Reef population, the values were significantly lower than zero, showing potential selective sweeps.

Furthermore, we identified selected genomic regions and genes of the introduced population of *P*. *fucata*. As shown in Supplementary Table S2, 448 regions were under selection, containing a total of 481 genes. Of these genes, 82 genes were among the top 5% in terms of *F_ST_* values, 436 genes were among the top 5% in terms of ROD values and 37 genes were among the top 5% of both *F_ST_* values and ROD values. Among the 481 genes, 416 genes were annotated to the Nr, KEGG or GO database (Supplementary Table S3). Ultimately, we found some notable genes, including the heat shock transcription factor 1 gene, an endoglucanase gene, a sodium channel protein genes and others (Supplementary Table S2).

## 4. Discussion

In recent decades, numerous invasions of bivalve species have occurred worldwide due to their high fecundity, rapid growth, high filtration rates, early sexual maturation and interactions with human activities [39,40]. The activities of introduced bivalve species, for instance, shell production, bioturbation and filter feeding, may change ecosystem processes and functions and affect biodiversity and the environment [7,41]. Therefore, bivalve invasions have become a serious ecological problem around the world.

The process of invasion can be divided into three distinct phases: initial introduction, establishment and spread in the introduced range [42]. As shown in our investigation, *P*. *fucata* in Mischief Reef is now in the establishment phase. Although the population does not currently cause any ecological problems in Mischief Reef, *P*. *fucata* has dispersed beyond its original distribution range. It could be considered a Stage III invasive species in Mischief Reef according to the definition of Collauti and MacIsaac, due to fact that the *P. fucata* population has the potential to establish itself in the Mischief Reef [43]. 

### 4.1. Population Genetic Structure and the Potential Origin of the Mischief Reef Population

In the present study, we analysed the population genetic structure of three native *P. fucata* populations and an introduced nonlocal *P. fucata* population. Our work aimed to detect the potential origin of the Mischief Reef population and assess the genetic status of the introduced population. In our study, we selected Lingshui and Zhanjiang populations, because ships or humans from these two sites display more intensive activities. Thus, we considered that the two sites were the potential original source. As shown in Figure 2B, the Mischief Reef population and Lingshui population were clustered into a group, indicating a closer relationship than that of Mischief Reef population with others. Furthermore, both the topology of the NJ phylogenetic tree and the *F_ST_* values clearly provided evidence for the close relationship of the Mischief Reef and Lingshui populations. Takeuchi et al. [44] indicated that the western Pacific population could be divided into a southern population (China) and a northern population (Japanese mainland). Overall, the *F_ST_* values were low, supporting the results of a previous study [45]. Nevertheless, the *F_ST_* values between Lingshui and Mischief Reef were significantly lower than those between other pairs of populations in our study, suggesting that the Lingshui population may be a potential original resource of the introduced population in Mischief Reef. In the structure analysis, *K* = 2 was identified as the most suitable number of clusters. Unfortunately, under the suggested cluster number, only Haiphong could be identified from the mixed population. Then, some clusters coherent with geographical location emerged when the cluster number was increased. The Zhanjiang population first separated from the remaining mixed populations when the cluster number was three. Nevertheless, the Mischief Reef population could not be separated clearly from the Lingshui population when the cluster number was increased continually. Furthermore, the results of gene flow analysis showed a high gene flow between Lingshui and Mischief Reef populations. Overall, all the results of population genetic structure supported the hypothesis that the population in Mischief Reef was potentially introduced from Lingshui.

Previous studies have shown that detecting the precise origin of an introduced population can be extremely challenging [16,45]. In addition to effective approaches, a large area sampling effort is also required to ensure that the real origins are included [16]. Furthermore, numerous studies have indicated that multiple introductions and multiple origins might be a common phenomenon in most invasions [46]. However, the introduced population in Mischief Reef was less likely to have multiple origins, partly because of political sensitivity. Moreover, in the Mischief Reef population, we did not observe higher genetic variation caused by multiple introductions. Due to the limited sampling size and area, our study has limitations and we could not precisely detect the origin of the Mischief Reef population. However, we reported the presence of the *P. fucata* population in Mischief Reef in the South China Sea for the first time and provided references for further studies reconstructing its introduction history and novel insights into oyster invasions.

### 4.2. Population Genetic Diversity and LD in P. fucata Populations

In the present study, the nucleotide diversity (*θ_π_*) of the *P. fucata* populations at the whole-genome level was 0.0034–0.0051, indicating significantly higher genetic diversity than in other studies [47]. Huang et al. [48] estimated that cultured *P. fucata* populations in Lingshui and Zhanjiang showed expected heterozygosity values of 0.2636 and 0.2374, respectively. In our study, the expected heterozygosity of the four populations was 0.2377–0.2622. Thus, the genetic diversity of the four populations in our study was moderate. However, the Mischief Reef population showed lower nucleotide diversity than the other populations in the present study. Notably, our data clearly revealed lower genetic diversity in the introduced population than in the potential original population. This result is consistent with previous studies of introduced populations [16]. It is well known that introduced populations experience the loss of genetic variation during the founding event as a result of founder effects and bottlenecks [43,49,50].

As a critical parameter in population genetics, LD could provide deep insight into the complex population history, including selection, hybridization and mutation, of the introduced population during invasion [50]. Unexpectedly, we did not observe a dramatic increase in LD in the introduced population with respect to the native *P. fucata* populations. Furthermore, relatively rapid LD decay was observed in the Mischief Reef population. Previous studies indicated that a dramatic increase in LD was a common phenomenon in most crop domestication and some invasive organisms [51,52]. We speculated that there were abundant individuals that contributed to the introduction and funding of the Mischief Reef population. Therefore, an increase in LD was not found in the present study. On the other hand, an LD decrease can be attributed to population size and recombination rate [53]. Some researchers have demonstrated that introduced populations may undergo a high level of recombination with mutation as a result of rapid population expansion [50,54]. Hence, we attribute the LD decrease to the rapid expansion of the Mischief Reef population. Furthermore, since the LD decay in the introduced population was rapid, we suspect that the population in Mischief Reef may not have undergone strong natural selection during founding.

### 4.3. Potential Selected Regions in the Introduced Population

The allele frequency and nucleotide diversity differentiation between populations provide important genetic evidence of selection processes [55]. Meanwhile, Tajima’s *D* represents the frequency of rare variants at a genomic locus. These parameters indicate whether the regions and genes are under selection. In our study, the Tajima’s *D* values of nonnative population and its potential origins were negative and significantly lower than those of other native populations. The results indicated an excess of rare nucleotide site variants compared to the expectation under a neutral model of evolution [56].

Furthermore, we found some genes that were under selection in the introduced population, including the heat shock transcription factor 1 gene, an endoglucanase gene, a serine/threonine-protein phosphatase gene, a sodium channel protein and other genes. However, due to the limited number of selected genes, enrichment pathway analysis could not be performed. According to previous studies, salinity and sea surface temperature are the major factors affecting the distribution of *P. fucata* populations [44,57]. The selected genes are related to temperature and salinity tolerance. Thus, we speculate that these genes play important roles in adaptation to the different environment of Mischief Reef, such as higher salinity and more stable temperature compared with those in Lingshui.

## 5. Conclusions

In the present study, we analysed the population genetic structure and diversity of three native *P. fucata* populations and an introduced nonlocal *P. fucata* population. Our results revealed a closer relationship between the Mischief Reef introduced population and the Lingshui population, indicating that Lingshui may be the potential geographical origin. Furthermore, our data revealed lower genetic diversity in the introduced population than in the potential original population. In the introduced population, we also identified some selected genomic regions and genes related to temperature and salinity tolerance. Our study will facilitate further aquatic conservation and protection in Mischief Reef, South China Sea. 

## Figures and Tables

**Table 1:** Population genetic parameters of the four populations.

	*H_e_*	*H_o_*	*θ_w_*	*θ_π_*	Tajima’s *D*
Mischief	0.2377	0.0942	0.0037	0.0034	−0.2932
Zhanjiang	0.2403	0.0967	0.0044	0.0045	0.0347
Lingshui	0.2502	0.1233	0.0049	0.0045	−0.3231
Haiphong	0.2622	0.1556	0.0048	0.0051	0.2054

**Table 2:** Fixation index (*F_ST_*) between different populations.

	Hainan	Mischief Reef	Zhanjiang
Lingshui			
Mischief	0.048		
Zhanjiang	0.069	0.095	
Haiphong	0.087	0.118	0.102

## Data Availability

All RAD-seq data in the present study were deposited in NCBI. The accession numbers and more details can be found in the Supplementary Table S4.

## Supplementary Materials

The following supporting information can be downloaded at: https://www.mdpi.com/article/10.3390/biology12010097/s1, Figure S1: The occurrence of *P. fucata* in Mischief; Figure S2: Cross-validation (CV) error calculated by structure analysis; Table S1: Clean reads statistics; Table S2: Genomic regions and genes under selection of Mischief population; Table S3: The information of potential selected genes; Table S4: The accession information of RAD-seq data in the present study.
