# Supplementary material for: Genetic Signature of Pinctada fucata Inferred from Population Genomics: Source Tracking of the Invasion in Mischief Reef of Nansha Islands
Source: Biology (Basel). 2023 Jan 9;12(1):97. doi: 10.3390/biology12010097 (PMC9855575; doi:10.3390/biology12010097)
Supplement: Supplementary file 1 [file biology-12-00097-s001.zip › Supplementary Figures---Pinctada fucata.pdf]

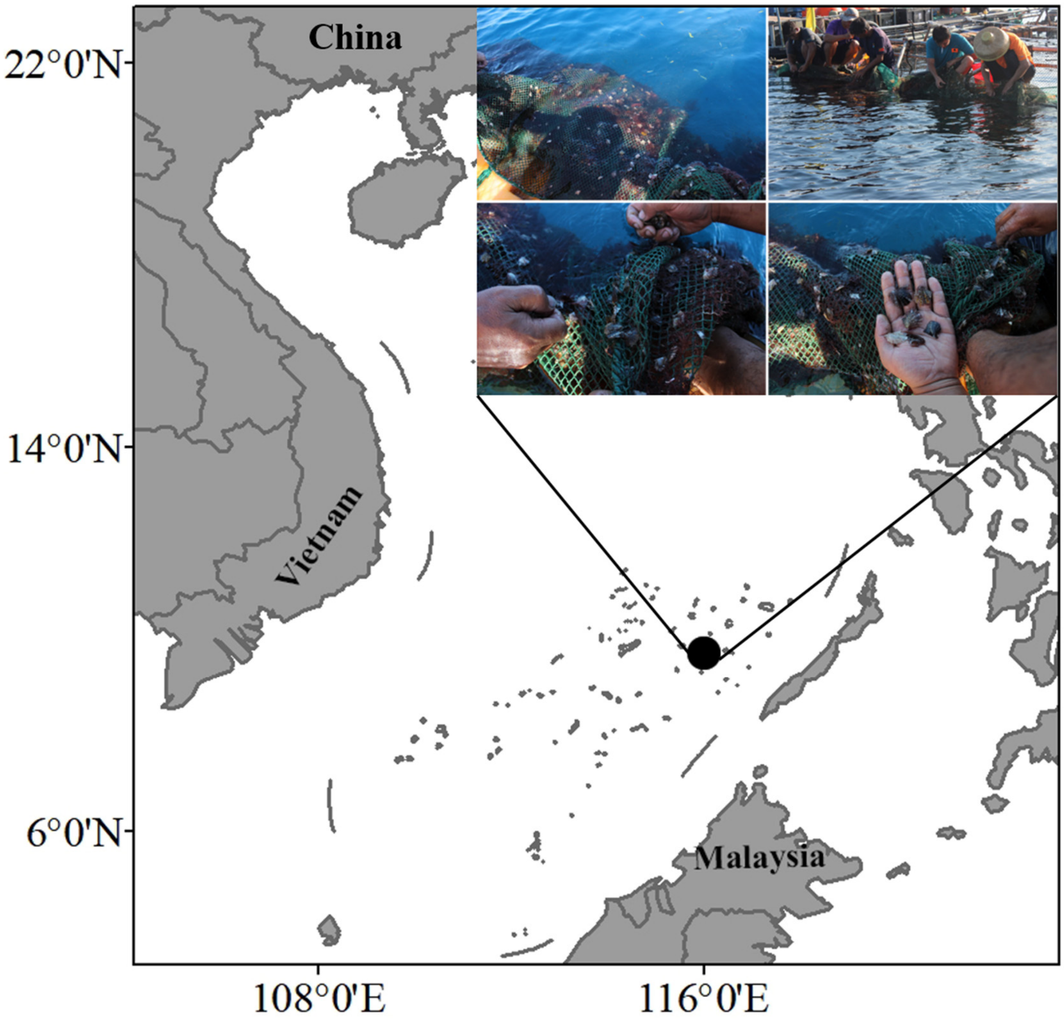

Figure S1. The occurrence of *P. fucata* in Mischief.

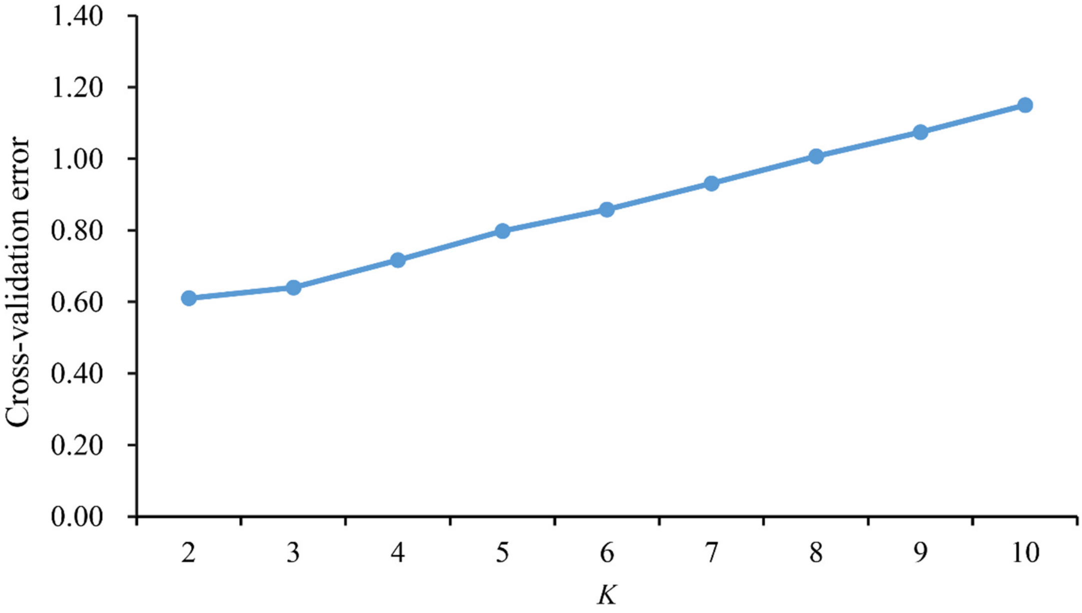

Figure S2. Cross-validation (CV) error calculated by structure analysis.
